# Liquid Chromatography Mass Spectrometry-Based Metabolite Pathway Analyses of Myeloma and Non-Hodgkin’s Lymphoma Patients

**DOI:** 10.22074/cellj.2017.4412

**Published:** 2017-05-17

**Authors:** Carl Angelo D. Medriano, Jinhyuk Na, Kyung-min Lim, Jin-ho Chung, Youngja H. Park

**Affiliations:** 1 Metabolomics Laboratory, College of Pharmacy, Korea University, Sejong City, Korea; 2 College of Pharmacy, Ewha Woman’s University, Seoul, Korea; 3 College of Pharmacy, Seoul National University, Seoul, Korea

**Keywords:** Multiple Myeloma, Non-Hodgkin’s Lymphoma, Mass Spectrometry

## Abstract

**Objective:**

This study attempted to identify altered metabolism and pathways related to non-Hodgkin’s lymphoma (NHL) and myeloma patients.

**Materials and Methods:**

In this retrospective study, we collected plasma samples from 11 patients-6 healthy controls with no evidence of any blood cancers and 5 patients with either multiple myeloma (n=3) or NHL (n=2) during the preliminary study period. Samples were analyzed using quadrupole time-of-flight liquid chromatography mass spectrometry (LC-MS). Significant features generated after statistical analyses were used for metabolomics and pathway analysis.

**Results:**

Data after false discovery rate (FDR) adjustment at q=0.05 of features showed 136 for positive and 350 significant features for negative ionization mode in NHL patients as well as 262 for positive and 98 features for negative ionization mode in myeloma patients. Kyoto Encyclopedia of Genes and Genomes (KEGG) pathway analysis determined that pathways such as steroid hormone biosynthesis, ABC transporters, and arginine and proline metabolism were affected in NHL patients. In myeloma patients, pyrimidine metabolism, carbon metabolism, and bile secretion pathways were potentially affected by the disease.

**Conclusion:**

The results have shown tremendous differences in the metabolites of healthy individuals compared to myeloma and lymphoma patients. Validation through quantitative metabolomics is encouraged, especially for the metabolites with significantly expression in blood cancer patients.

## Introduction

Blood cancers are a heterogeneous disease group that affect the body’s hematopoietic and lymphatic tissue (1). These cancers have higher incidence rates in men and evidence suggests that immunosuppression, infections, ultraviolet radiation, chemical exposures, and genetic susceptibility are involved in their pathogenesis (2). Among these cancers are non-Hodgkin’s lymphoma (NHL) and multiple myeloma. NHL are malignancies that arise from the lymphoid tissue, often with various clinical and biological features. According to histologic characteristics, NHL is divided into B- and T-cell neoplasms, especially the lymphocyte development stage and are classified additionally into clinical features (3). On the other hand, multiple myeloma is a malignancy of the plasma cells which results in an overproduction of monoclonal immunoglobulins (4). Similar to NHL, the pathogenesis of this cancer is poorly understood but there are insights that link its clinical entity with the cancer’s etiology (5).

Patients with diffuse large B-cell lymphoma (DLBCL), the most common lymphoid malignancy, rely on a regimen of cyclophosphamide, doxorubicin, vincristine, and prednisone (CHOP) as the main standard of care. This regimen has cured 35% of patients in phase 2 studies (6). A more intensive strategy which involves high-dose chemotherapy followed by autologous stem-cell transplantation (ASCT) has been found to cure nearly half of the patients with chemotherapy-relapsed DLBCL (7). Recently, analysis of biologic heterogeneity has focused on individual genes that emphasized those with treatment outcome, known function on other malignancies, and normal lymphocyte development (8). Other than the possibility of highlighting potential pathogenic mechanisms of the disease, comprehensive molecular signatures of tumors might identify promising targets for therapeutic intervention (9, 10). Another therapeutic option for NHL patients, radioimmunotherapy (RIT), uses a monoclonal antibody coupled with a radionuclide to deliver radiation to the diseased sites. RIT has been extensively studied with encouraging results. Meanwhile, advances in multiple myeloma in clinical practice have reached a deeper understanding of the biology of its clone and interaction with the bone-marrow microenvironment where it resides (11). Being able to recognize the importance of the tumor microenvironment is one of the most important progresses in the field of this malignancy which has helped improve treatment options (12). However, patients encounter challenges such as drug resistance and toxicity after receiving treatment with available medications. Approximately 40% of patients who received bortezomib (1.3 mg/m^2^) twice weekly complained of peripheral neuropathy (13). On the other hand, novel therapies which include modulators of protein homeostasis, immunomodulatory agents, kinase inhibitors, targeting accessory cells and cytokines, and immune-based therapies appeared promising in the field of therapeutic development for multiple myeloma patients (14). Although these therapies involve improvement of existing treatment regimens or changing cellular targets, they have not considered other viewpoints such as metabolism of affected patients for possible relevance to drug efficacy, treatment failure, relapse of disease and other complications. Therefore, there is a need to recognize other methods that utilize advanced high-throughput technologies to address this particular concern.

Although systems biology taught us that genome, transcriptome, and proteome are important, metabolome is still considered to be the most representative of the phenotype (15). Thus, understanding the human cancer metabolome may be the best way to reveal phenotypic changes relative to biological functions, especially where metabolite concentrations can easily be traced (16). The introduction of metabolomics as an emerging technology in cancer research has led to useful information in the aspects of cancer metabolism, specifically for the central mechanisms in tumors. High resolution metabolomics (HRM) is popular in different fields of study due to its accurate and unbiased measurement of metabolites from organisms that use sensitive technologies such as nuclear magnetic resonance spectroscopy (NMR) and liquid chromatography mass spectrometry (LC-MS) (17, 18). LC-MS HRM can measure low molecular compounds in samples such as extracts, blood, and urine (19, 20). This technique is widely used because of its potential aid to understand the effects on different processes or pathways with the help of different databases such as Metlin (21) and Kyoto Encyclopedia of Genes and Genomes (KEGG) (22, 23). A previous study has applied this technique in the discovery of novel biomarkers related to lung cancer (24). In this preliminary study on metabolic profiling of blood cancer patients in South Korea, we evaluated the differentially expressed metabolites found in two hematologic malignancies-multiple myeloma and NHL using LC-MS-based metabolomics from plasma of patients. This study aimed to use HRM to identify affected pathways linked to these diseases which might open alternative viewpoints on the management and treatment of these blood cancers.

## Materials and Methods

In this retrospective study, we collected plasma samples with consent from 11 patients after approval by the Ethics Committee of Health Service Center at Seoul National University. There were 6 healthy controls (without blood cancers) and 5 patients with either multiple myeloma (n=3) or NHL (n=2) during the preliminary study period.

### Quadrupole time-of-flight liquid chromatography mass spectrometry

We treated 50 μl sample aliquots with acetonitrile (1:2, v/v), after which they were centrifuged at 14000 x g for 5 minutes at 4˚C to remove proteins (25). Samples were analyzed in triplicate using quadrupole time-of-flight LC-MS (Agilent, Santa Clara, CA, USA) for both positive and negative electrospray ionization modes. High-resolution metabolomics is mostly used to analyze highly complex metabolite mixtures since detection of mass/ charge (m/z) with 10 ppm or better mass resolution as well as mass accuracy substantially decreases the need for physical separation of metabolites prior to detection. Detection from 50 to 1000 m/z of ions at 20000 resolution over a 35-minute LC run with data extraction using apLCMS provides a minimum of 3000 reproducible features, many with sufficient mass accuracy to allow prediction of elemental composition (26). An m/z feature is defined by m/z, ion intensity, and retention time.

### Metabolic profiling with univariate and multivariate statistical analysis

Data analyses were carried out using all technical replicates. The raw data was processed with apLCMS which produced the total features of the samples needed for statistical analysis. These features were averaged, log2 transformed and quantile normalized before we applied bioinformatics and statistical analyses which included univariate analysis, the Manhattan plot, and false discovery rate (FDR) adjustment to determine the significant metabolites between those with hematologic malignancies and healthy individuals (27). Thereafter, metabolic profiles were discriminated using Limma-hierarchical clustering analysis (HCA) to separate two groups in association with the differentiated metabolites. Limma is originally a package for the analysis of gene expression data that arises from microarray or RNA-Seq technologies. It provides the ability to make simultaneous comparisons between numerous targets (28, 29).

### Data annotation and pathway analysis

The significant features were annotated using Metlin database (https://metlin.scripps.edu/) (25). The data from Metlin includes potential identity of the compound, mass, chemical formula, and KEGG numbers which are used to map these metabolites in a human metabolic pathway map found online at www.genome.jp/kegg/. This database predicts the number of potentially affected metabolites and pathways from the significant features identified and gives a visualization that may vary depending on the studied organism. Various types of information regarding reactions, network, and interactions is found in the KEGG pathway (30).

## Results

Patient’s demographics are shown in Table 1.

**Table 1:** Patient demographics


	Controln=6Mean ± SD	Myeloman=3Mean ± SD	NHLn=2Mean ± SD

Age (Y)	48.7 ± 7.8	54.7 ± 8.7	52 ± 28.3
Female gender	33%	33%	0%
WBC (thousands/µL)		10.29 ± 5.63	7.41 ± 1.68
RBC (millions/µL)		3.05 ± 0.29	3.14 ± 0.41
Hemoglobin (g/dL)		9.67 ± 0.35	9.35 ± 1.27
Hematocrit (%)		29.5 ± 1.3	30 ± 2.83
Platelets (thousands/µL)		114.33 ± 31.56	305 ± 98.99
Lymphocytes (%)		25 ± 21.07	4.9 ± 2.69


NHL; Non-Hodgkin’s lymphoma, WBC; White blood cell, and RBC; Red blood cell.

Statistical analysis of age difference showed that the age differences of control and patients was not significant (P>0.05).

### Manhattan plot and two-way hierarchical clustering analysis

**Fig.1:**
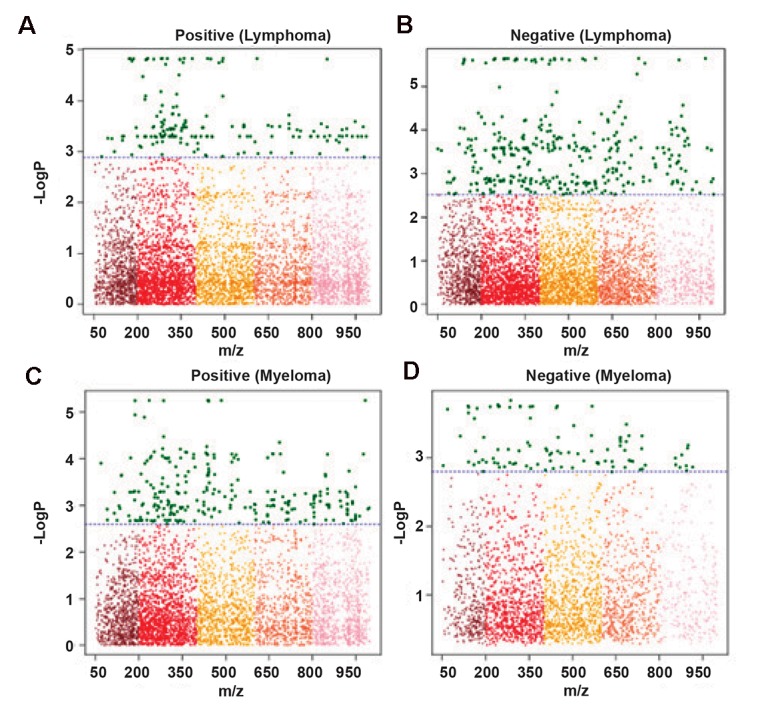
Manhattan plot of the significant features found in non-Hodgkin’s lymphoma (NHL) patients for A. Positive, B. Negative ionization modes, and multiple myeloma patients for C. Positive, and D. Negative ionization modes.

We used the metabolome-wide association study (MWAS) to identify changes in the concentrations of metabolites from the plasma of control (without blood cancers) and case (lymphoma and myeloma) patients. Figure 1 shows the Manhattan plot of the significant features of lymphoma and myeloma patients for both positive and negative ionizations after FDR adjustment with a threshold of q=0.05. Of the 17282 aligned features from apLCMS in the positive ionization mode of the QTOF LC/MS, there were a total of 163 significant features for lymphoma and 262 significant features for myeloma. The negative ionization mode which aligned 11829 features showed 350 significant features for NHL and 98 significant features for myeloma. These significant m/z features were used to identify metabolites for pathway analysis. Manhattan plot combines statistical analyses (e.g., P value, ANOVA) with the magnitude of change and enables visual identification of statistically significant data-points (metabolites, etc.) that display large-magnitude changes. Multiple testing corrections such as FDR adjusts P values (q-values) derived from multiple statistical tests correct the occurrence of false positives. The y-axis of this plot represents the negative log of the raw P value that compared the concentration of each metabolite in plasma between healthy controls and blood cancer patients. The x-axis were the m/z values that ranged from 50 to a maximum of 1000 to satisfy the condition where only compounds which fell into this range could be considered. The dashed line on the graph showed the FDR adjustment made in the data to eliminate false positives, therefore m/z values above this line were significantly expressed from the ones below this line (Fig.1).

**Fig.2:**
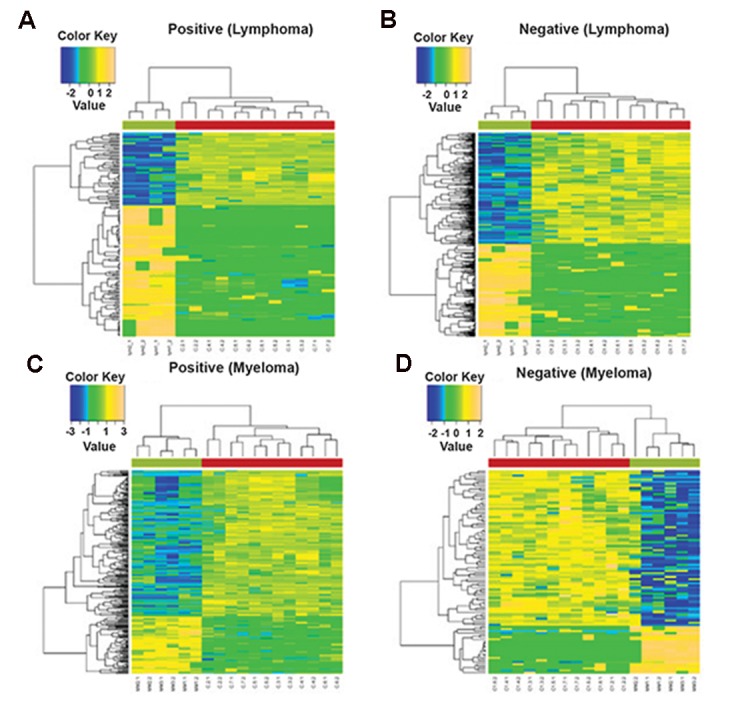
Hierarchical clustering analysis (HCA) of non-Hodgkin’s lymphoma (NHL) patients for A. Positive, B. Negative ionization modes, and multiple myeloma patients for C. Positive, and D. Negative ionization modes.

We performed two-way HCA analysis on the significant metabolite features to identify the essential metabolites for sample clustering. In this study, HCA used the 163 (positive) and 350 (negative) significant features from NHL and 262 (positive) and 98 (negative) from myeloma patients which were the key components that separated cancer patients and healthy individuals. In HCA, the significant features are grouped depending on their correlation (e.g, signal intensities). HCA determines the similarity measures using Euclidean distance and Pearson linear correlation The panels on top of the figures have shown two distinguishable main clusters for both positive and negative ionization modes-green for NHL and myeloma patients, while control samples were grouped in the red panels (Fig.2). The clear separation of the significant features between control and case samples could be regarded as an indication of the differences in expression of metabolites in malignant patients compared to healthy individuals. The colors of the metabolites represented their relative concentration which was based on the signal intensities from LC-MS data. This indicated that the concentration of the metabolites from blood cancer patients was significantly higher or lower than the control samples, depending on which color spectrum they fell under.

### Identification of significant metabolites in blood cancer patients

Metlin generates matrices that contain information such as mass, adduct, name, chemical abstracts service (CAS) number, and KEGG numbers of the metabolites specifically used for further processing of data in pathway analysis. These metabolites have been filtered at a confidence limit of 30 ppm which told the database to list down all possible metabolites that fall up to ± 0.003% m/z difference from the indicated mass. Metlin used the mass of the significant m/z features and select adducts to match metabolites according to their similarities to these specifications. In this study, we used the adducts [M+H]+and [M+Na]+for positively ionized metabolites as well as [M+Cl]-and [M-H]-for negatively ionized metabolites due to their abundance in the human body. For NHL patients, we identified 300 metabolites from the 163 significant features in the positive ionization mode and 832 metabolites from 350 features in the negative ionization mode. Meanwhile, 305 metabolites out of 262 significant features from the positive mode and 190 metabolites from 98 significant features from the negative mode of myeloma patients were identified. Table 2 lists some of these compounds.

The identities of these metabolites are questionable and need further verification. The most common way to validate a metabolite’s identity and concentration in the samples is through quantitative analysis by NMR and MS. This study, however, has not validated the significant metabolites in NHL and myeloma patients. This can be a focus by future studies that use metabolomics of hematologic malignancies. The complete annotated metabolites for both ionization modes of myeloma and NHL patients are shown in the Supporting Information. Their roles on the metabolism of humans will be discussed in the next section.

### Human metabolic pathway map analysis using the Kyoto Encyclopedia of Genes and Genomes

**Fig.3:**
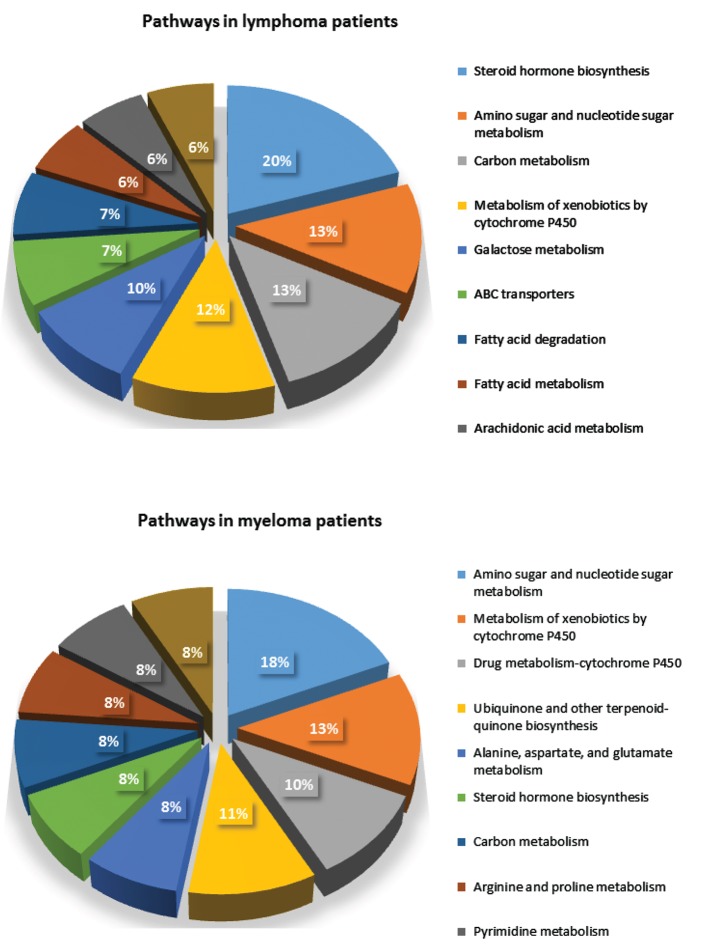
Ten pathways with the highest numbers of affected metabolites for non-Hodgkin’s lymphoma (NHL) and myeloma patients from Kyoto Encyclopedia of Genes and Genomes (KEGG) pathway analysis.

**Fig.4:**
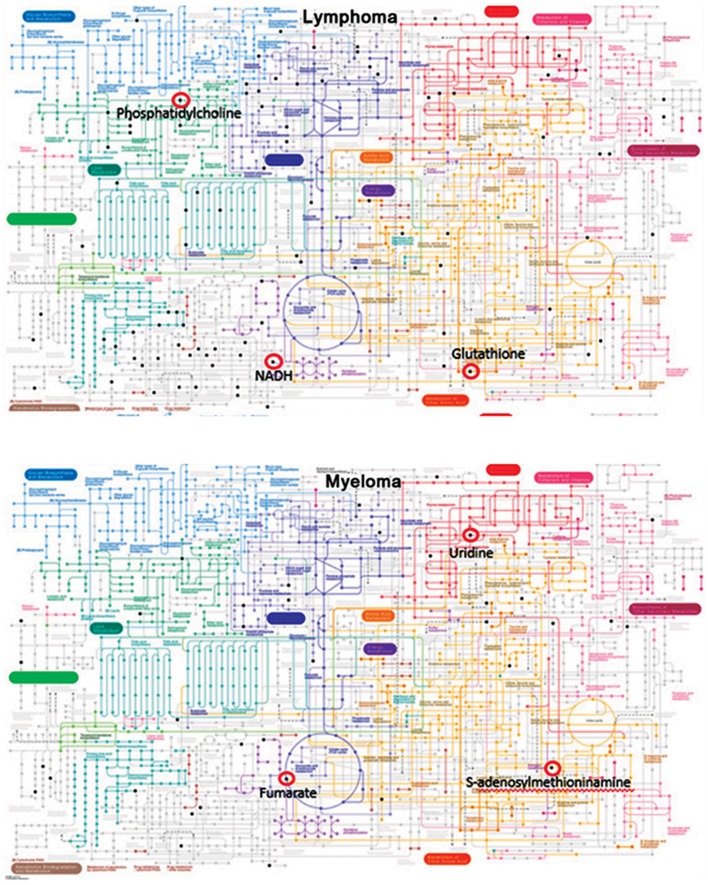
Metabolic pathway map of humans that show some of the affected metabolites in non-Hodgkin’s lymphoma (NHL) and myeloma patients.

The metabolites with KEGG numbers were used in the metabolic pathway analysis of blood cancer patients. The resulting KEGG numbers from both the ionization modes were combined and analyzed together for an easier analysis. We observed a total of 90 compound hits that belonged to 100 metabolic pathways from NHL patients and 36 compound hits from 114 pathways in myeloma patients. Figure 3 shows the 10 pathways with the most affected metabolites. The values per partition of the pie graph show the percentage of the number of metabolites affected per pathway to the total number of metabolites in the 10 pathways listed. The complete list of metabolic pathways affected can be found in the Supporting Information. Aside from those listed in Figure 3, other pathways such as oxidative phosphorylation, choline metabolism in cancer, and bile secretion pathways with metabolites like NADH, phosphatidylcholine, and glutathione were observed to be affected in NHL patients. Meanwhile, for myeloma patients we have observed that fumarate, uridine, and S-adenosylmethioninamine (Fig.4) which are under the oxidative phosphorylation pathway, pyrimidine metabolism, and cysteine and methionine metabolism were some of the metabolites potentially affected by the disease.

**Table 2:** A number of identified metabolites from cancer patients annotated by using the Metlin database


Cancer	Ionization	Molid	Inputmass	Adduct	Mass	dppm	Name	Formula	CAS	KEGG

Lymphoma	Positive	134	181.0710326	[M+H]^+^	180.0634	2	D-Galactose	C6H12O6	59-23-4	C00124
	Negative	193	339.2059151	[M+Cl]^-^	304.2402	10	Arachidonic Acid (peroxide free)	C20H32O2	506-32-1	C00219
Myeloma	Positive	60264	856.7260108	[M+H]^+^	855.7081	12	Phosphatidylcholine	C50H98NO7P		C00157
	Negative	66524	141.0463524	[M+Cl]^-^	106.0783	9	p-Xylene	C8H10	106-42-3	C06756


CAS; Chemical abstracts service and KEGG; Kyoto Encyclopedia of Genes and Genomes.

## Discussion

In this study, we observed a clear separation of metabolites between healthy controls and patients with myeloma and NHL, which indicated a difference in metabolites expressed in patients with these cancers. We identified these significant features by the untargeted metabolomics approach in order to assess differences in multiple metabolic pathways affected in NHL and myeloma patients. Lodi et al. (31) used the same approach to evaluate metabolic profiles of patients with myeloma during diagnosis, post-treatment remission, and disease progression by proton NMR-based metabolite analysis. Similarly, Yoo et al. (32) identified hypoxanthine from urine samples as a marker for NHL by untargeted low-mass ion profiling. Meanwhile, this study has analyzed plasma samples from healthy individuals as well as those from myeloma and NHL patients to check for diverse pathways which are affected but may be neglected in myeloma and NHL studies. Although identification of potential biomarkers for these diseases is important, recognizing which pathways are generally affected is equally essential in understanding the effects of these malignancies on human metabolism.

The pathways and those in the Supporting Information were pathways where the significantly expressed metabolites in myeloma and NHL patients belonged. Oxidative phosphorylation modulation by mitochondria is believed to regulate tumor growth in cancer (33). NADH-linked decreased oxidation of substrates, overproduction of reactive oxygen species, and altered control of apoptosis are a number of metabolic changes presented by many cancer cells (34). Abnormal choline metabolism is a new prospect to study metabolism related to oncogenesis and tumor progression. Clinically, cancers that develop in different organs demonstrate elevated total choline levels. Magnetic resonance studies of choline phospholipid metabolism in cancer have confirmed that these choline compounds are elevated in cancers (35, 36). Meanwhile the role of the bile secretion pathway in blood malignancies, which has a known function of absorption and metabolism of fats as well as fat-soluble vitamins in the small intestine, has yet to be explored. One of the metabolites affected in this pathway, glutathione, is a major cellular antioxidant crucial for maintaining the balance between oxidation and reduction. It is also important in cellular detoxification and immune response (37). In contrast, some studies have found that elevated levels of glutathione in tumors may increase resistance to chemotherapy and radiotherapy (38, 39). Although this pathway and metabolites are associated with different cancers, they have not been fully explored in the diagnosis, management, or treatment of NHL. In multiple myeloma patients, fumarate is an intermediate in the citric acid cycle used by cells to produce energy in the form of ATP. Elevated intracellular fumarate together with inhibition of fumarate hydratase (FH) coincides with hypoxia-inducible factor (HIF) upregulation, thus permitting tumorigenesis (40). This phenomenon has been seen in renal cancer cells (41). Another seemingly important metabolite in cancer under the pyrimidine metabolism is uridine. This natural pyrimidine nucleoside is one of the most promising biological modulators for anticancer drug (e.g., 5-fluoroacil) efficacy to solid tumors which has been seen in preclinical models (42, 43). To date, an association between S-adenosylmethioninamine and cancer has not been determined. Its decarboxylated form, S-adenosylmethionine, is increasingly recognized for its role in hepatocyte growth, death, and malignant degeneration (44). Similar to the pathways and metabolites found in lymphoma patients, these metabolites have not been related to myeloma as far as the authors’ knowledge is concerned.

Pathway analysis is important in understanding metabolic transitions following cellular transformation. This can lead to new insights into the biological basis of transformation and may generate novel targets for therapy and cancer diagnosis (45). Perroud et al. (46) utilized pathway analysis using proteomics and metabolic profiling to discover highly significant pathways for renal cell carcinoma. The group also featured the metabolic changes related to kidney cancer and its applicability for optimal therapy. Despite the benefits of this technology, to the best of our knowledge, pathway analysis has not been used for studies of both myeloma and NHL patients. KEGG pathway analysis also shows affected metabolites using the metabolic pathway map where the metabolites are highlighted (e.g., black) in small nodes. The colored nodes represent various metabolic pathways in different organisms which is easily differentiated by color intensity-dark-colored pathways are from the chosen organism while light-colored pathways belong to other organisms. The researchers believe that the metabolites and pathways discussed in this study will enable future studies in blood cancers to investigate new possibilities in improving current knowledge of these malignancies for better management and treatment options. The application of HRM, not only in this study, but also in other cancer researches paves the way to a further understanding of its possible interactions which may eventually lead to improved diagnosis or may aid in the development of a more effective treatment.

## Conclusion

This study discussed the metabolic profiling of two blood malignancies-NHL and myeloma. We used LC-based metabolomics to identify all possible affected pathways and metabolites from the plasma samples of healthy and cancer patients. There was a clear metabolic difference observed from the NHL and myeloma samples compared to the healthy controls. Affected pathways, like oxidative phosphorylation and choline metabolism, were those linked in tumor growth and progression. The pathways and metabolites discussed, despite their indirect association with these hematologic malignancies, might open various possibilities in management and treatment options for the patients. Regardless of the small number of samples due to the lack of NHL and myeloma patients, the samples used in this study were analyzed in replicates and have undergone strict statistical analyses for a more reliable data set. Future studies that involve more patient samples are recommended to verify and strengthen the findings in this study. 
